# Nuclear factors involved in mitochondrial translation cause a subgroup of combined respiratory chain deficiency

**DOI:** 10.1093/brain/awq320

**Published:** 2010-12-17

**Authors:** John P. Kemp, Paul M. Smith, Angela Pyle, Vivienne C. M. Neeve, Helen A. L. Tuppen, Ulrike Schara, Beril Talim, Haluk Topaloglu, Elke Holinski-Feder, Angela Abicht, Birgit Czermin, Hanns Lochmüller, Robert McFarland, Patrick F. Chinnery, Zofia M.A. Chrzanowska-Lightowlers, Robert N. Lightowlers, Robert W. Taylor, Rita Horvath

**Affiliations:** 1 Mitochondrial Research Group, Institute for Ageing and Health, Newcastle University, Newcastle upon Tyne, NE4 5PL, UK; 2 Medical Genetic Centre, Munich, Germany; 3 Department of Paediatric Neurology, University of Essen, Germany; 4 Department of Paediatrics, Paediatric Pathology Unit, Hacettepe University, Ankara, Turkey; 5 Institute of Human Genetics, Newcastle University, Newcastle upon Tyne, NE1 3BZ, UK

**Keywords:** mitochondrial translation, combined respiratory chain deficiency, early-onset encephalomyopathy

## Abstract

Mutations in several mitochondrial DNA and nuclear genes involved in mitochondrial protein synthesis have recently been reported in combined respiratory chain deficiency, indicating a generalized defect in mitochondrial translation. However, the number of patients with pathogenic mutations is small, implying that nuclear defects of mitochondrial translation are either underdiagnosed or intrauterine lethal. No comprehensive studies have been reported on large cohorts of patients with combined respiratory chain deficiency addressing the role of nuclear genes affecting mitochondrial protein synthesis to date. We investigated a cohort of 52 patients with combined respiratory chain deficiency without causative mitochondrial DNA mutations, rearrangements or depletion, to determine whether a defect in mitochondrial translation defines the pathomechanism of their clinical disease. We followed a combined approach of sequencing known nuclear genes involved in mitochondrial protein synthesis (*EFG1, EFTu, EFTs, MRPS16, TRMU*), as well as performing *in vitro* functional studies in 22 patient cell lines. The majority of our patients were children (<15 years), with an early onset of symptoms <1 year of age (65%). The most frequent clinical presentation was mitochondrial encephalomyopathy (63%); however, a number of patients showed cardiomyopathy (33%), isolated myopathy (15%) or hepatopathy (13%). Genomic sequencing revealed compound heterozygous mutations in the mitochondrial transfer ribonucleic acid modifying factor (*TRMU*) in a single patient only, presenting with early onset, reversible liver disease. No pathogenic mutation was detected in any of the remaining 51 patients in the other genes analysed. *In vivo* labelling of mitochondrial polypeptides in 22 patient cell lines showed overall (three patients) or selective (four patients) defects of mitochondrial translation. Immunoblotting for mitochondrial proteins revealed decreased steady state levels of proteins in some patients, but normal or increased levels in others, indicating a possible compensatory mechanism. In summary, candidate gene sequencing in this group of patients has a very low detection rate (1/52), although *in vivo* labelling of mitochondrial translation in 22 patient cell lines indicate that a nuclear defect affecting mitochondrial protein synthesis is responsible for about one-third of combined respiratory chain deficiencies (7/22). In the remaining patients, the impaired respiratory chain activity is most likely the consequence of several different events downstream of mitochondrial translation. Clinical classification of patients with biochemical analysis, genetic testing and, more importantly, *in vivo* labelling and immunoblotting of mitochondrial proteins show incoherent results, but a systematic review of these data in more patients may reveal underlying mechanisms, and facilitate the identification of novel factors involved in combined respiratory chain deficiency.

## Introduction

Combined respiratory chain deficiency characterizes a subset of mitochondrial diseases exhibiting decreased activities of multiple complexes of the oxidative phosphorylation system, leading to an impairment of ATP synthesis (DiMauro and Schon, 2003; Smits *et al.*, 2010*a*). Combined respiratory chain deficiency has previously been associated with mitochondrial DNA rearrangements (e.g. Kearns–Sayre syndrome) that affect mitochondrial transfer RNA and/or ribosomal RNA genes (Tuppen *et al.*, 2010) leading to an overall decrease in respiratory complex function via defective gene transcription and translation, or single mitochondrial transfer RNA point mutations resulting in dysfunctional translation of multiple mitochondrial respiratory complex subunit genes (Hsieh *et al.*, 2001; Karadimas *et al.*, 2001).

Mitochondrial DNA depletion causes an overall reduction in respiratory competency of the affected cell or tissue (Barthelemy *et al.*, 2001; Sarzi *et al.*, 2007). Most patients with mitochondrial DNA depletion carry autosomal recessive mutations in nuclear genes participating in mitochondrial DNA replication, in the balanced supply of deoxynucleotide triphosphates to mitochondria or a component of the mitochondrial replisome (Spinazzola *et al.*, 2009). Mitochondrial DNA depletion is a frequent cause of severe childhood (hepato)encephalomyopathies and is responsible for ∼50% of combined respiratory chain deficiencies in childhood (Sarzi *et al.*, 2007).

Nuclear DNA mutations can account for combined respiratory chain deficiency by negatively affecting mitochondrial maintenance, translation and/or transport. It has been hypothesized that defective nuclear genes, which function in mitochondrial translation, are the primary cause of combined respiratory chain deficiency in patients that present with neither mitochondrial DNA mutations nor mitochondrial depletion (Jacobs and Turnbull, 2005; Smits *et al*., 2010*b*).

Miller *et al*. (2004) identified the first human disease related to a nuclear-encoded impairment of mitochondrial protein synthesis caused by a homozygous nonsense mutation in the ribosomal protein gene *MRPS16* (NG_008096.1; GI:193082974). Pathogenic mutations of another mitoribosomal protein gene, *MRPS22* (NG_012174.1; GI:237874203), have also been reported in severe antenatal-onset infantile disease (Saada *et al*., 2007). The notion that combined respiratory chain deficiency was correlated to a mutation in a nuclear gene affecting mitochondrial translation prompted further functional studies of mitochondrial translation in patients with combined respiratory chain deficiency, which resulted in the identification of mutations in mitochondrial translation elongation factor genes *EFG1* (*GFM1*; NG_008441.1; GI:197333723), *EFTu (TUFM*; NG_008964.1 GI:212549715), *EFTs (TSFM*; NG_016971; GI:62531056) and *C12orf65* (NG_027517.1, GI: 304361771) (Coenen *et al.*, 2004; Antonicka *et al.*, 2006, 2010; Smeitink *et al.*, 2006; Valente *et al.*, 2007). Mutations in mitochondrial transfer RNA modifying factors may also impair mitochondrial translation, as in myopathy, lactic acidosis and sideroblastic anaemia syndrome, a rare condition associated with mutations in the pseudouridylate synthase 1 gene (*PUS1*; NM_025215.5; GI:259155298; Bykhovskaya *et al*., 2004; Fernandez-Vizzara *et al.*, 2007). Very recently, mutations were described in patients with myopathy, lactic acidosis and sideroblastic anaemia syndrome in the mitochondrial tyrosyl transfer RNA synthetase gene (*YARS2*; NC_000012.11; GI:224589803; Riley *et al.*, 2010). Mutations in nuclear genes encoding the mitochondrial aspartyl (*DARS2*; NG_016138.1; GI:270289741) and arginyl (*RARS2*; NG_008601.1; GI:201862389) transfer RNA synthetases were also described in very characteristic neurological phenotypes, such as leucoencephalopathy with brainstem and spinal cord involvement (Scheper *et al.*, 2007; Isohanni *et al.*, 2010) and cerebellar and vermian hypoplasia (Edvardson *et al.*, 2007); however, not all these patients presented with combined respiratory chain deficiency. Recently, autosomal recessive mutations in the mitochondrial transfer RNA modifying enzyme (*TRMU*; NG_012173.1; GI:237874202) were described in infantile reversible hepatopathy (Zeharia *et al.*, 2009) and were also reported to modify the phenotype of the mitochondrial DNA mutation m.1555A>G (Guan *et al.*, 2006), opening up the possibility that mitochondrial transfer RNA modifying factors may play an important role in patients with combined respiratory chain deficiency.

To date, the number of patients identified as harbouring mutations in nuclear genes affecting mitochondrial translation is very limited (Table 1). To define whether combined respiratory chain deficiency is attributed to nuclear defects in mitochondrial translation in patients, we took two parallel approaches: (i) sequencing the genomic DNA of known human candidate genes and (ii) *in vivo* metabolic ^35^S-methionine labelling of mitochondrial protein synthesis and immunoblotting for two mitochondrial-encoded and one nuclear-encoded proteins in human primary cells.

**Table 1:** Clinical presentation of previously described patients with combined respiratory complex deficiency and mutations in nuclear genes affecting mitochondrial protein synthesis

**Gene (number of cases)**	**Family history**	**Age at symptom onset/death**	**Clinical presentation**					**Additional symptoms**	**Histology RRF/ COX- fibres/other**	**Reference**
			**M**	**N**	**H**	**L**	**LA**			
Nuclear components of the mitochondrial translation machinery										
*EFG1* (2)	Consanguinity, affected sibling	Birth/27 d	+	+	–	+	+	Intrauterine growth retardation, corpus callosum hypoplasia, cystic brain lesion	Normal muscle	Coenen *et al.*, 2004
*EFG1* (2)	Affected sibling	Birth/9 d	+	+	DA	+	+	Intrauterine growth retardation, dysmorphy	Many COX-fibres, no RRF	Antonicka *et al.*, 2006
*EFG1* (1)		3 w/16 m	+	+	–	–	+	Dysmorphy, microcephaly	SDH+/COX-fibres, lipid accumulation	Valente *et al.*, 2007
*EFTu* (1)		2 d/14 m	+	+	+/–	+	+	Macrocystic leukodystrophy, polymicrogyria	nd	Valente *et al.*, 2007
*EFTs* (1)	Consanguinity	Birth/ 7 w	+	+	DA	–	+	Rhabdomyolysis, epilepsy	nd(?)	Smeitink *et al.*, 2006
*EFTs* (1)	Consanguinity	2 d/7 w	+	–	+	–	+	Low urinary output, hyponatraemia	Generalized COX-	Smeitink *et al.*, 2006
*C12orf65* (3)	Consanguinity	1 y/>22 y	+	+	–	–	+	Leigh syndrome, optic atrophy, ophthalmoplegia	nd	Antonicka *et al.*, 2010
Ribosomal protein genes										
*MRPS16* (1)	Consanguinity	1 d/9 d	+	+	DA	+	+	Corpus callosum agenesia, dysmorphy	nd	Miller *et al.*, 2004
*MRPS22* (2)	Consanguinity, affected sibling	Birth/22 d	+	–	+	–	+	Subcutaneous oedema, tubulopathy	nd	Saada *et al.*, 2007
tRNA modifying genes and tRNA synthetases										
*PUS1* (6)	Consanguinity 2 families		+	–	–	–	+	Severe sideroblastic anaemia, mental retardation, dysmorphic features	Mitochondrial myopathy	Bykhovskaya *et al.*, 2004
*PUS1* (2)	Consanguinity, affected sibling	6 m/12 y	+	–	–	–	+	Growth retardation, severe sideroblastic anaemia, cognitive impairment, dysmorphy	COX-/RRF, myopathy	Fernandez-Vizarra *et al.*, 2007
*RARS2* (3)	Consanguinity	Birth/16 m	+	+	–	–	+/–	Cerebellar and vermian hypoplasia. microcephaly	nd	Edvardson *et al.*, 2007
*DARS2* (several)	Consanguinity		–	+	–	–	–	Leucoencephalopathy with brainstem and spinal cord involvement	Normal?/nd	Scheper *et al.*, 2007
*DARS2* (8)			–	+	–	–	–	Leucoencephalopathy with brainstem and spinal cord involvement	Normal?/nd	Isohanni *et al.*, 2010
*TRMU* (13)	Consanguinity	2–4 m	–	–	–	+	+	Isolated reversible hepatopathy	Normal muscle, mitochondrial pathology in liver	Elpeleg 2009
*YARS2* (3)	Consanguinity	10 w/>24 y	+	–	+	–	+	Severe sideroblastic anaemia, cardiomyopathy	RRF/COX-fibres	Riley *et al.*, 2010

DA = open ductus arteriosus; H = heart disease; L = liver involvement; LA = lactic acidosis; M = muscle; N = neurological symptoms; d = day; w = week; y = year; m = month; RRF = ragged red fibres; nd = not determined.

## Materials and methods

### Patients

We have investigated 52 patients with combined respiratory chain deficiency, where mitochondrial DNA rearrangements, depletion and point mutations were excluded as the underlying cause of the disease. These patients were collected in two core mitochondrial diagnostic centres (Newcastle and Munich) over >15 years. The clinical presentation, histological and biochemical findings of the patients are summarized in Table 2. DNA samples and primary cell cultures (15 myoblast and 7 fibroblast cell lines, see Table 2) of these patients were analysed in this study. Informed consent was obtained from all participants in accordance with protocols approved by local institutions.

**Table 2:** Summary of the clinical presentation of 52 patients with combined respiratory complex deficiency

**Patient/gender**	**Family history**	**Age at symptom onset/death**	**Clinical presentation**				**Additional symptoms**	**Muscle histochemistry**			**Respiratory chain deficiency**
			**muscle**	**CNS**	**heart**	**liver**		**SDH^+^**	**COX^–^**	**Other**	
P1/F		5 w/4 m	+	–	–	–	LA	–	–		I + IV
P2/M	Consanguinity	4 m/8 m	+	–	–	–	Respiratory failure	–	–	Lipid	I + III + IV
P3/M	Consanguinity	2 w/3 w	+	–	–	–	LA	–	++		I + III + IV
P4/F	Consanguinity, 2 siblings died	1 m/1 m	+	–	+	–		–	+++		I + IV
P5/M		24 d	+	+	–	–		+	–		III + IV
P6/F		18 m	+	+	–	–	CC agenesia	–	–		I + III + IV
P7/M	Consanguinity, 2 affected siblings	15 d	+	–	–	–	Respiratory failure	–	++	Lipid	I + III + IV
P8/F	Consanguinity	Birth	+	–	+	–	LA	–	++		II/III + IV
P9/M		13 y	+	–	–	–		–	+++	Inflammation	I+III+IV
P10/F	Consanguinity	3 w/2 m	+	–	+	–	LA	++	++		I + III + IV
P11/F	Consanguinity, 1 affected sibling	12 m	+	–	+	–		+	–		I + IV
P12/F		3 y	+	+	–	–	LA	+	–		I + IV
P13/F		10 y	+	+	–	+	int.pseudoobstr, cataract	–	–	Lipid	I + IV
P14/M		3 m	+	–	–	–		+	–		I + III + IV
P15/M		Birth	+	+	–	–	small ASD	+	–		I + III
P16/F		1 y	+	+	–	–	LA	–	–	Lipid	I + II/III
P17/F		Birth/4 m	+	–	+	–	LA	+	–		I + IV
P18/F	Consanguinity	Birth	+	+	+	–	long-chain acyl-carnitine↑	–	–	Lipid	I + III, mild IV
P19/M		Birth	+	+	–	–	arthrogryposis, CC agenesia, dysmorphy, deafness	+	–		I + IV
P20/M		4 y	+	+	–	–	Ophthalmople-gia, LA basal ganglia lesion	–	–		I + IV
P21/M		Birth/3 d	+	+	–	–		–	++		I + IV
P22/M		15 m	+	+	–	+	Respiratory failure	–	+		I + IV
P23/F		12 m	+	+	–	–	Cellular immundefect	nd	nd	nd	I + IV
P24/M	Consanguinity	4 y	+	+	–	–	LA	–	–		I + IV
P25/F	Consanguinity	Birth	+	+	–	–	LA, coma	–	+		I + IV
P26/M		Birth	+	–	–	–	Floppy baby	+	–		I + IV
P27/M		Birth	+	+	+	–		–	+		I + III + IV
P28/F		10 y	+	+	–	–	Basal ganglia calcification	–	+		I + IV
P29/F	Consanguinity	2 y	+	+	–	–		nd	nd	nd	I + III
P30/F	Affected twin sister	2 y	+	+	–	+		–	–		I + IV
P31/F	Consanguinity	4 y	+	+	–	–		++	–		I + IV
P32/F	Consanguinity, affected sibling, cousin	10 m	+	+	–	–	Optic atrophy, brain atrophy	–	–		I + II/III
P33/F		2 y/3 y	+	+	–	+	Demyelination on autopsy	+	–		I + IV (liver)
P34/F	Consanguinity, affected sibling	Birth/1 m	+	+	–	–	LA	–	–		I + IV
P35/F		Birth/3 w	+	–	+	–	LA	++	+++		I + IV
P36/M	Consanguinity, affected brother	18 m/2 y	+	–	–	+		+	–		I + IV (liver)
P37/F		18 m	–	–	–	+	Reversible disease	+	–		I + IV (muscle and liver)
P38/M	Consanguinity	16 y	+	+	–	–	Diabetes, myoclonic jerks	–	++		I + IV
P39/M		<1 y	–	+	–	+	CC agenesia, leukodystrophy	–	+		I + IV
P40/F		<1 y	+	–	–	–	Respiratory failure	–	+++		I + III + IV
P41/F		<1 y	+	+	–	–	bilateral. IVH,SH	–	–		I + III + IV
P42/M		1–2 y?	+	+	+	–		–	+++		I + IV
P43/M		4 m/8 m	+	–	+	–		–	+++		I + IV
P44/F		<1 y	+	–	+	–		–	–	Lipid	I + III + IV
P45/M	Consanguinity	<1 y	+	+	–	–	Deafness, renal tubular acid.	–	+++		I + IV
P46/M		9 y	+	+	–	–	Deafness, bulbar symptoms	–	–		I + IV
P47/M		3 y	+	+	+	–	Metabolic acidosis	nd	nd	nd	I + IV
P48/F	Consanguinity, affected sister	nd	+	–	+	–		–	++		I + III + IV
P49/M		birth/<1 y	+	–	+	–		–	+++		I + III + IV
P50/F		<1 y	+	+	+	–	LA	nd	nd	nd	I + IV
P51/M		birth	+	–	+	–		–	+++		I + III + IV
P52/M		27 y	+	+	+	–	Pancytopenia	–	+++		I + IV

+++ = severe (>25%); ++ = moderate (5–25%); + = mild (<5%).

ASD = atrial septal defect; CC = corpus callosum; IVH = intra-ventricular haemorrhage; LA = lactic acidosis; mt = mitochondria; nd = not determined; SH = subdural haemorrhage; d = day; w = week; y = year; m = month.

### Muscle histology and biochemistry

Muscle biopsies from all patients were investigated using standard histological and histochemical assessments. Cryostat sections (10 µm) were stained for cytochrome *c* oxidase (COX), succinate dehydrogenase (SDH) and sequential COX–SDH double histochemical staining to identify COX-deficient fibres (Taylor *et al.*, 2004). The activities of respiratory chain complexes I–IV were determined and corrected for citrate synthase activities in skeletal muscle and/or liver as described earlier (Fischer *et al.*, 1986; Tulinius *et al.*, 1991; Kirby *et al.*, 2007).

### Fibroblast and myoblast tissue culture

Primary cell cultures from 22 of the 52 patients and also from four controls were obtained from the Biobank of the Medical Research Council Centre for Neuromuscular Diseases, Newcastle and the Muscle Tissue Culture Collection, Munich (Table 2). Fibroblasts were grown in minimal essential medium (Life Technologies, Paisley, UK), supplemented with 10% foetal calf serum, 1% glutamine, 100 mg/ml streptomycin, 100 U/ml benzylpenicillin, 110 µg/ml sodium pyruvate and 50 µg/ml uridine. Muscle cells were grown in skeletal muscle growth medium (PromoCell, Heidelberg, Germany), supplemented with 4 mM l-glutamine and 10% foetal bovine serum and cultured as recommended by the supplier.

### DNA analysis

Genomic DNA was isolated from either primary cell lines or from muscle biopsies, using the DNeasy® Blood and Tissue kit (Qiagen, Valencia, CA, USA). Genetic analysis for mitochondrial DNA rearrangements and mitochondrial depletion was performed by standard methods (Murphy *et al.*, 2008). Direct sequencing of the entire mitochondrial genome was undertaken using muscle DNA as template, employing 36 pairs of M13-tagged oligodeoxynucleotide primers as described earlier (Taylor *et al.*, 2003). Amplified polymerase chain reaction products were sequenced using BigDye® Terminator v3.1 chemistries (Applied Biosystems) and compared with the revised Cambridge reference sequence (GenBank Accession number NC_012920) (Andrews *et al.*, 1999).

Cytogenetic analysis including comparative genomic hybridization array was performed in patients with dysmorphological signs or congenital abnormalities. Whole genomic DNA was labelled, sephadex G50 purified and hybridized together with human reference DNA (400 ng each) on bacterial artificial chromosome arrays (CytoChip V2.1, BlueGnome, Cambridge, UK) according to the manufacturer’s protocol.

The entire coding region of the genes *EFTs*, *EFG1* and *EFTu* was sequenced by using M13-tailed intronic primers, as described earlier (Valente *et al.*, 2007). We designed primers for sequencing *MRPS16* and *TRMU* (see online supplementary material). The analysis was performed on an ABI 3130xl sequencer and the data analysed using SeqScape program (Applied Biosystems). The impact of all identified non-synonymous amino acid substitutions on protein function were predicted using Sorting Intolerant From Tolerant software and by Alamut (Interactive Biosoftware, Rouen, France). All synonymous and intronic changes were analysed for the possibility of a splicing defect by Alamut.

### *In vivo* labelling and analysis of mitochondrial protein synthesis

*In vivo* ^35^S-methionine labelling studies were performed as described earlier (Chomyn *et al.*, 1996) with the following modifications. Cells, cultured to 60–70% confluency in T25cm^2^ flasks, were pretreated with Dulbecco’s modified Eagle’s medium (Sigma, Poole, UK) containing 10% (v/v) foetal bovine serum, 50 µg/ml uridine and 50 µg/ml chloramphenicol for 24 h at 37°C/5% CO_2_. Cells were subsequently washed with phosphate-buffered saline (Sigma, Poole, UK) and incubated for 15 min at 37°C/5% CO_2_ in methionine/cysteine and foetal bovine serum-free Dulbecco’s modified Eagle’s medium, supplemented with 5% (v/v) dialyzed foetal bovine serum, 0.1 mg/ml anisomycin (Sigma, Poole, UK). Following addition of 200 mCi/ml ^35^S-methionine/cysteine (^35^S EasyTag EXPRESS; Perkin Elmer, Beaconsfield, UK), cells were incubated for 2 h at 37°C/5% CO_2_, then washed with phosphate-buffered saline and a cell pellet prepared. Total protein yield was calculated by Bradford assay and equal quantities of total protein (50 μg) were pretreated with 1U Benzonase nuclease (Merck and Co., Inc, NJ, USA) for 1 h. Pretreated samples were then separated by sodium dodecyl sulphate–polyacrylamide gel electrophoresis. Radiolabelled proteins were visualized by PhosphorImager/ImageQuant analysis (Amersham Biosciences, Little Chalfont, UK). The identities of the mitochondrial-encoded oxidative phosphorylation complex gene products were identified in accordance with (Chomyn, 1996).

### Immunoblotting

Immunoblotting was performed in six patient cell lines (Patients 9, 10, 12, 19, 32 and 36) and three controls. Aliquots of total protein (5–20 µg) were loaded on 14% sodium dodecyl sulphate–polyacrylamide gels, transferred to polyvinylidene fluoride membranes and subsequently probed with monoclonal antibodies recognizing porin (Molecular Probes), mitochondrial COXI (Molecular Probes), COXII (Mitosciences) or CI-20/complex I subunit CI-20/NDUFB8 (Mitosciences) according to the recommendations of the suppliers. Following incubation with horseradish peroxidase-conjugated secondary antibodies (Dako, Denmark A/S) detected proteins were visualized by ECL-plus (GE Healthcare). COXI and COXII are mitochondrial-encoded proteins. The presence of NDUFB8 shows good correlation with the correct assembly of mitochondrial-encoded complex 1 subunits.

## Results

### Clinical presentation

The clinical presentation of the 52 patients with combined respiratory chain deficiency is summarized in Table 2. The vast majority (50/52; 96%) of our patients were children (age of onset <15 years), most of them (34/52; 65%) had an early onset of symptoms within the first year of life; however, there were two adults with onset at age 16 (Patient 38) and 27 (Patient 52) years. The mean age of onset was ∼2.5 years. The most frequent clinical presentation of children with combined respiratory chain deficiency––similar to other early-onset mitochondrial conditions––was muscular hypotonia and muscle weakness (50/52; 96%), accompanied by encephalopathy (33/52; 63%), cardiomyopathy (17/52; 33%) and hepatopathy (7/52; 13%). Some children showed very severe, early-onset multisystem phenotypes, with symptoms already at birth (13/52; 25%), in some infants accompanied by congenital developmental anomalies (5/52; 9.5%) and rarely with dysmorphological signs. All patients except two showed muscular hypotonia and weakness, but the involvement of other organs was more heterogeneous (encephalopathy, cardiomyopathy, hepatopathy). Two patients (4%) did not show muscular hypotonia, but a predominant liver dysfunction, either isolated (Patient 37) or in combination with encephalopathy (Patient 39). Some rare additional symptoms were also noted in a few cases such as agenesis of the corpus callosum (*n* = 3) or other brain MRI abnormalities [white matter lesions (*n* = 2), calcification of the nucleus caudatus (*n* = 1), basal ganglia lesions (*n* = 1) and severe cortical atrophy (*n* = 1)]. Deafness was reported in three patients. Cellular immunodeficiency, pancytopenia, arthrogryposis, renal tubular acidosis, optic atrophy and intestinal pseudo-obstruction were present in single patients only. Although there were some clinical similarities defined in small number of patients, such as patients with liver disease, cardiomyopathy or agenesis of the corpus callosum, there was a substantial overlap, making it difficult to form homogeneous phenotypic groups. Patient 37 had a characteristic clinical presentation of reversible isolated liver disease with no clinical involvement of skeletal muscle or any other tissue.

### Muscle histology and biochemistry

Muscle histochemistry showed COX-deficient and/or SDH hyper-reactive fibres in 33 patients (63%). SDH hyper-reactive fibres, suggesting mitochondrial proliferation, were noted in 14 patients (27%), COX-deficient fibres were detected in 22 patients (42%), and both SDH hyper-reactive and COX-deficient fibres were present in two patients only. Muscle histochemistry was normal in 15 patients (29%) indicating that these findings are frequent, but not necessarily present in all patients and normal mitochondrial histochemistry does not exclude combined respiratory chain deficiency. No correlation was found with the clinical phenotype. Lipid accumulation was detected in six patients, implying a possible link with lipid metabolism. Muscle histology and histochemistry data were not available in four patients.

All patients showed significant reduction in activity levels in more than one respiratory chain complex. Fifteen children (29%) had a defect of all mitochondrial DNA-encoded enzymes (I, III, IV), but with normal complex II activity clearly indicating a generalized problem of mitochondrial protein synthesis. Other patients showed decreased activities of two or more complexes in different combinations. The most frequent combination was I + IV defects, detected in 31 children (60%). Decreased activities of I + III or I + II/III––that may reflect a defect in coenzyme Q10 biosynthesis––were observed in four patients (8%). A defect of II/III + IV was found in two patients only. The biochemical defect was very severe (<10% residual activity of more than one respiratory chain enzyme) in nine patients (17%), most of whom had a severe early-onset multisystem phenotype; however, patients with a milder biochemical defect do not necessarily present with a less severe disease. In Patient 37, the respiratory chain deficiency was expressed in both liver and muscle, the latter tissue not being clinically affected, indicating that tissue-specific differences may occur.

### Genetic analysis

Mitochondrial DNA analysis was performed on all patient samples to exclude mitochondrial DNA deletions/depletion or pathogenic mutations. A homoplasmic *COI* mitochondrial DNA variant, m.7444G>A, was detected in Patient 9, which is most likely not pathological for the disease. However, no further possible disease causing mitochondrial mutations were identified in the remaining patients and no patients showed a significant reduction in mitochondrial copy number, thereby excluding mitochondrial DNA depletion. No chromosomal abnormalities were detected in any of the patients.

**Figure 1:**
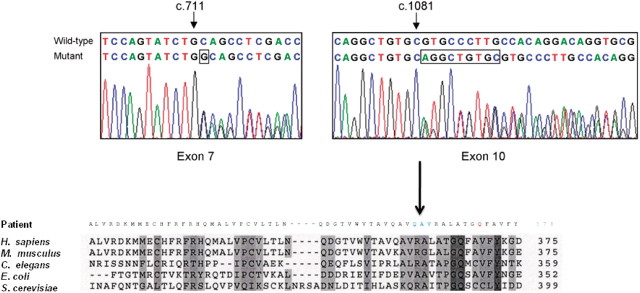
Sequence electropherograms of the compound heterozygous *TRMU* mutations in Patient 37 (*top*). Both wild type and mutant sequences are detailed. Inserted bases are highlighted (boxes). Alignment of C-terminal sequence of human *TRMU* with its homologs (*bottom*). Mutant sequence with the three amino acid insertion in blue and the highly conserved shifted glutamine residue in red are shown.

We detected two compound heterozygous *TRMU* mutations in Patient 37 (Fig. 1). The heterozygous 1 base pair insertion c.711_712insG causes a frameshift and premature stop after 252 amino acids (p.Gln238Ala fsX14), which is clearly pathogenic. This mutation was heterozygous in the healthy mother. The second mutation is a heterozygous nine base pairs in-frame insertion, c.1081_1082insAGGCTGTGC, causing the insertion of three amino acids in position p.361 (alanine, valine, arginine). This insertion neighbours a highly conserved glutamine residue, which has been shown to be involved in anticodon recognition, with mutation of this residue resulting in an inactive enzyme (Numata *et al.*, 2006). Presumably altering the position of this critical residue due to an insertion may inhibit the activity of TRMU. This variant was heterozygous in the healthy father. Both mutations were absent in 100 normal chromosomes of the same ethnic origin (German and British). In three additional patients (Patients 4, 27 and 49) we have detected another missense variant in *TRMU*, c.28G>T, p.Ala10Ser either in heterozygous (Patients 4, 27) or homozygous (Patient 49) state, which has been reported earlier in healthy controls (National Centre for Biotechnology Information, rs11090865). None of these patients had hepatopathy.

By sequencing the other nuclear genes that might affect mitochondrial protein synthesis, we identified a total of three single heterozygous non-synonymous nucleotide changes in three different genes (c.34T > C, p.Tyr12His in *MRPS16;* c.860T > A, p.Leu287His in *EFTs;* c.1990G>A, p.Val664Ile in *EFG1*) in three independent patients, but all three changes are listed in the National Centre for Biotechnology Information database as single nucleotide polymorphisms.

### *In vivo* labelling and analysis of mitochondrial translation products

**Figure 2:**
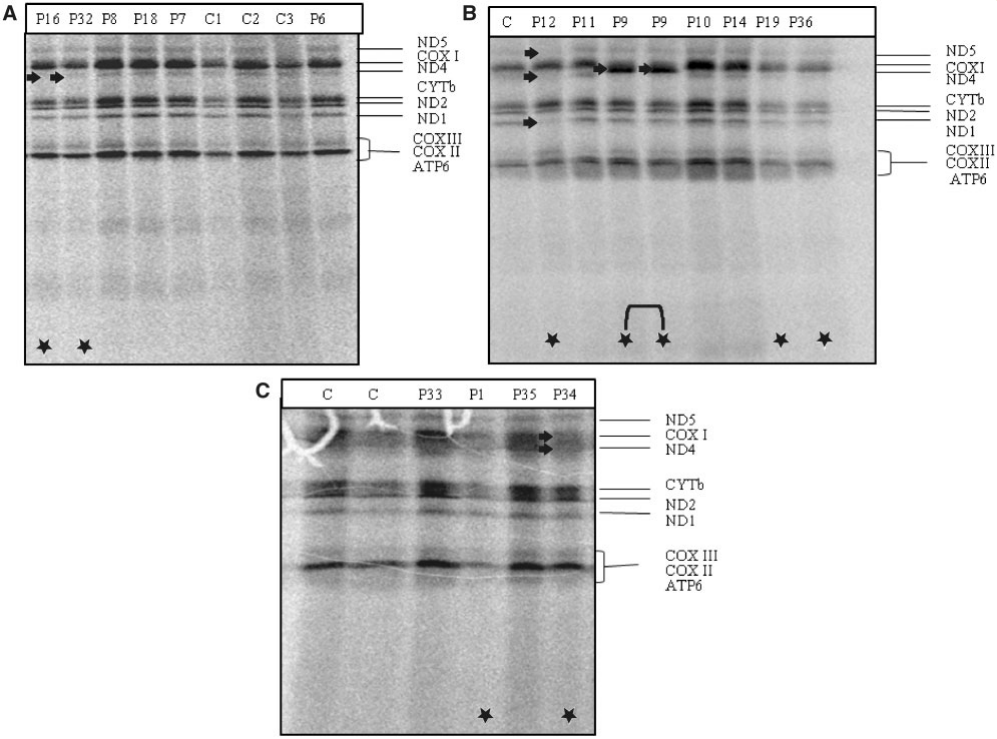
^35^S-methionine pulse labelling of myoblast (**A** and **B**) and fibroblast (**C**) cell lines. Patients (P) 1, 19 and 36 showed a significant overall reduction in mitochondrial oxidative phosphorylation complex subunits. Patients 32 and 16 showed an isolated decrease in the translation of ND4. Patient 12 had reduced steady-state levels of ND1, ND4 and ND5, with normal ND2, and COXI and ND4 were decreased in Patient 34. Patient 9 contained a structurally altered, smaller COXI mitochondrial protein. Deficient bands and lanes are marked with white arrows and stars.

Three of the 22 patients' cells (Patients 1, 19 and 36) showed a significant overall reduction in mitochondrial oxidative phosphorylation complex subunit protein abundance, when compared with the control samples (Fig. 2), whereas selective impairment was found in four additional patients (Patients 12, 16, 32 and 34). To exclude loading or pipetting problems, these results were confirmed with different loading concentrations by repeated analysis. Although mitochondrial DNA copy number was within normal range in these patients’ muscle DNA, we cannot completely exclude the possibility that the *in vivo* labelling result in these patients is related to a decreased amount of mitochondria.

Two cell lines showed an isolated decrease in the translation of NADH dehydrogenase (ND) subunit 4 (Patients 16 and 32), suggesting that either the translation of ND4 is more sensitive on ^35^S-methionine labelling assay, or an isolated translation deficiency of this subunit is present; however, the combined respiratory chain defect in the patients’ muscle is supportive of the first explanation. Another patient had reduced steady state levels of ND1, ND4 and ND5 subunits with normal ND2 (Patient 12), which may reflect an isolated complex I related problem; however, both complex I and IV were decreased on biochemical measurement of respiratory chain enzymes in this patient’s skeletal muscle (Table 2). Myoblasts of Patient 9, carrying the homoplasmic mitochondrial DNA variant m.7444G>A contained a smaller COXI mitochondrial protein, which is presumably due to the homoplasmic *MTCOI* mitochrondrial DNA variant and is suggestive of the potential perturbation of the protein structure, and not for defective translation.

The translation products of COXI and ND4 were decreased in Patient 34, which may reflect the impairment in the protein synthesis of both complexes I and IV. Interestingly, a relatively high number of cell lines showed normal or even stronger than normal labelling (Fig. 2). Unfortunately, we did not have cells from the patient carrying *TRMU* mutations (Patient 37).

### Immunoblotting

**Figure 3:**
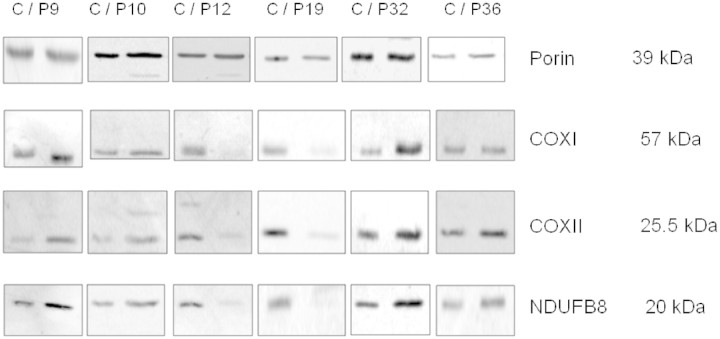
Immunoblotting for mitochondrial proteins COXI, COXII, NDUFB8 (representing ND subunits) and porin as a control protein in patients’ primary cell lines (P) compared with controls (C). Patients 12 and 19 showed an overall decrease of all three mitochondrial respiratory chain proteins COXI, COXII and NDUFB8. In Patients 10, 32 and 36 we detected normal and even stronger than normal signals for COXI, COXII and NDUFB8. In Patient 9, we could confirm the aberrant migration of COXI whilst the other mitochondrial-encoded proteins were normal.

Immunoblotting with monoclonal antibodies against the mitochondrial-encoded COX (COXI, COXII) and complex I subunits (nuclear-encoded NDUFB8, indicative for ND proteins) confirmed the overall decrease detected in translation assay in Patient 19 (Fig. 3; Table 3). The reduced bands for all three mitochondrial proteins in Patient 12 suggest that the decreased translation products of ND1, ND4 and ND5 on pulse labelling may reflect a translation defect of not only complex I, but also COX-related proteins, as was suggested by the combined respiratory chain defect in this patient’s skeletal muscle (Table 2), However, in contrast with the overall repression of mitochondrial translation in Patient 36 and the isolated decrease in translation of ND4 in Patient 32, we detected normal and even stronger than normal signal on immunoblotting with antibodies against COXI, COXII and NDUFB8 (Fig. 3; Table 3). In Patient 9, carrying the mitochondrial DNA variant m.7444G>A, consistent with the translation result, we confirmed the aberrant migration of COXI whilst the other mitochondrial-encoded proteins were normal (Fig. 3; Table 3). In Patient 10, both mitochondrial translation and protein levels were higher than normal, indicating a possible compensatory mechanism.

**Table 3:** Correlation between ^35^S-methionine pulse labelling, immunoblotting and respiratory complex activities in six patients

**Patient**	**^35^S-methionine pulse labelling**	**Immunoblotting**	**Respiratory complex activity**	**Muscle histochemistry**	
				**SDH^+^**	**COX**^–^
P9	COXI faster migration	COXI truncated band	I + II/III + IV ↓	–	+++
P10	Normal or overall ↑	COXI, COXII, NDUFB8 ↑	I + III + IV ↓	++	++
P12	ND1, ND4, ND5 ↓	COXI, COXII, NDUFB8 ↓	I + IV ↓	+	–
P19	Overall ↓	COXI, COXII, NDUFB8 ↓	I + IV ↓	+	–
P32	Only ND4 ↓	COXI, COXII, NDUFB8 ↑	I + II/III ↓	–	–
P36	Overall ↓	COXI, COXII, NDUFB8 ↑	I + IV ↓ (liver)	+	–

## Discussion

Combined respiratory chain deficiency is relatively common in mitochondrial encephalomyopathies and accounts for ∼30% of all respiratory chain deficiencies (Smits *et al.*, 2010*a*, *b*). Based on our experience in mitochondrial diagnostics in both children and adults, ∼40% of all combined respiratory chain deficiencies can be explained by mitochondrial DNA deletions and point mutations in mitochondrial transfer RNA genes, more frequently affecting adult patients. However, single and multiple deletions do not necessarily cause a biochemical respiratory chain deficiency. Approximately 40% of combined respiratory chain deficiency related to mitochondrial DNA depletion, affecting mostly young children with variable clinical presentation, is caused by autosomal recessive mutations in ≥9 nuclear genes (*DGUOK, MPV17, POLG, TYMP, TK2, SUCLA2, SUCLG1, RRM2B, PEO1*) influencing mitochondrial DNA replication and maintenance (Spinazzola *et al.*, 2009). In the remaining ∼20% of combined respiratory chain deficiencies, after excluding mitochondrial DNA deletions, depletion and point mutations, no clear diagnostic pathway is currently available to determine the cause of disease. The combinations of enzyme deficiencies may indicate that certain pathways are involved in the primary disease mechanism, although it is more likely to reflect a defect of the overall mitochondrial protein synthesis. In support of the second possibility, in patients with single mitochondrial DNA rearrangements or mitochondrial transfer RNA mutations, the respiratory chain deficiency may not affect all enzymes with mitochondrial-encoded subunits (frequently isolated complex I or I+IV defect, Tuppen *et al.*, 2010).

We analysed 52 patients with combined respiratory chain deficiency from two mitochondrial diagnostic centres. These individuals underwent thorough mitochondrial investigations, but the underlying cause was not identified. We focused our studies on investigations of mitochondrial translation, to define the frequency of a defective protein synthesis in combined respiratory chain deficiency.

The clinical, histological and biochemical data of our patients (Table 2) show similar clinical presentations (mitochondrial encephalomyopathy, cardiomyopathy, isolated myopathy, hepatopathy or multisystem disease) with the previously described patients listed in Table 1. The vast majority of our patients were children (age of onset <15 years) and most frequently had an early-onset of symptoms <1 year of age (65%). Both in our collective and in previously published reports a few children had a very severe, early-onset multisystem disease accompanied by congenital developmental anomalies and/or dysmorphological signs, which are rare presentations in other types of mitochondrial disease and may reflect a possible antenatal disease manifestation. It is also possible that some nuclear defects of mitochondrial translation are intrauterine lethal.

We identified seven patients (15%) with an isolated muscle involvement. The onset of symptoms was in most of these patients at a very early age (first weeks or months of life) except for one child who started to have muscle weakness at age 13 years. Because of the similarities to a characteristic clinical syndrome, infantile reversible COX deficiency myopathy, it is important to note that previous mitochondrial DNA sequencing excluded the m.14674T>C mutation in all these patients as an underlying cause of the disease (Horvath *et al.*, 2009). In two adult patients, an isolated myopathy with a probable muscle-specific defect of mitochondrial translation was reported by Sasarman *et al.* (2002). However, the primary genetic cause of the symptoms was not identified. Our patients with an isolated myopathy and combined respiratory chain deficiency further support the probability of a nuclear genetic factor controlling mitochondrial translation only in skeletal muscle.

The candidate gene approach had a very low detection rate, the primary causative mutations were detected in one patient only (1/52, 2%). In Patient 37 with isolated, reversible hepatopathy, we identified compound heterozygous mutations in the *TRMU* gene. This child (Patient 37) has a very similar clinical presentation to the previously published patients with *TRMU* mutations (Zeharia *et al.*, 2009), suggesting that *TRMU* mutations may lead to a clinically recognizable liver-specific phenotype and should be screened in all patients with early-onset hepatopathy. The possibility of a spontaneous recovery underlies the clinical importance in detecting these patients in an early phase of disease. We have detected another missense variant in *TRMU*, c.28G>T, p.Ala10Ser. This mutation in homozygous form has been described to influence the phenotypic presentation of the mitochondrial m.1555A>G mutation (Guan *et al.*, 2006) and was detected compound heterozygous with another pathogenic mutation in a child with reversible liver disease (Zeharia *et al.*, 2009). We have detected this variant more frequently in the diagnostic work-up of patients without clinical relevance and it is also present in controls (National Centre for Biotechnology Information, rs11090865), therefore, we think that this mutation, even if homozygous, is not sufficient to cause a severe phenotype, as observed in our patients. None of the three patients carrying the p.Ala10Ser mutation in either homozygous or heterozygous form had liver problems.

The tissue-specific presentation of our patients indicate, as with mitochondrial DNA depletion, that the phenotype may be representative of the genetic defect; however, we cannot exclude that mutations in several different genes may result in a similar phenotype. Complementation studies in patient cells would provide a useful tool to further investigate these possibilities (Murano *et al.*, 1997; Sasarman *et al.*, 2002).

We investigated the frequency of translational repression in 22 cell lines of patients with combined respiratory chain deficiency by ^35^S-methionine pulse labelling. Pulse labelling studies detected a defective mitochondrial translation in seven cell lines (32%), thereby indicating that a nuclear defect of mitochondrial protein synthesis may cause combined respiratory chain deficiency in about one-third of patients, without mitochondrial DNA abnormalities. However, the primary genetic cause was not detected by candidate gene sequencing in any of the seven patients. Other undefined molecular mechanisms are responsible for the combined respiratory chain deficiency in about two-thirds of patients. Three cell lines showed an overall decline in mitochondrial translation (3/22, 13%), a selective defect of the translation of different mitochondrial proteins was noted in four additional patients, indicating that a defect of mitochondrial translation may not affect all mitochondrial proteins. A selective defect of mitochondrial protein synthesis of the COXI subunit was recently identified in patients carrying pathogenic mutations in the *TACO1* gene (Weraarpachai *et al.*, 2009), underlying the possibility that nuclear genes may selectively affect the translation of different mitochondrial proteins.

A relatively high number of cell lines showed normal or enhanced pulse labelling, implying that either the combined respiratory chain deficiency is tissue specific and not expressed in cell culture, or related to factors downstream of protein synthesis. We performed preliminary experiments to examine the oxidative capacity of five cell lines that showed different patterns on ^35^S-methionine pulse labelling for mitochondrial translation (Patients 9, 10, 12, 32, 36 and control cells). This detected decreased levels of endogenous oxidative capacity in all cell lines, indicating the presence of a respiratory chain deficiency. It is important to note that recent reports have indicated normal or relatively unaffected levels of *de novo* synthesis of mitochondrial proteins, even after depletion of essential proteins involved in mitochondrial translation, including the release and recycling factors mitochondrial translational release factor 1 and mitochondrial ribosome recycling factor (Soleimanpour-Lichaei *et al.*, 2007; Rorbach *et al.*, 2008). Thus, it should not be overlooked that cells with severe respiratory defects may show apparently normal levels of ^35^S methionine labelling. Other explanations may be that the translated protein is unstable and triggers a compensatory increase in transcription and translation. This data may also reflect that in some patients, despite decreased translation, there is sufficient protein for immunodetection, although it is possible that the post-translational processing is altered. However, the respiratory chain activities were decreased in muscle, possibly localizing the defect downstream from mitochondrial translation (e.g. instability of proteins or altered function).

In a large collective of patients with combined respiratory chain deficiency we did not identify disease-causing mutations in *EFG1*, *EFTs*, *EFTu* and *MRPS16*, highlighting the difficulty of genetic diagnosis in these patients when using a candidate gene approach. The only patient with identified pathogenic mutations had a characteristic clinical presentation (reversible liver disease) that guided the genetic analysis and led to the identification of mutations in *TRMU*. In addition to *TRMU* mutations, deficiency of other mitochondrial transfer RNA-modifying proteins (transfer RNA pseudouridine synthase A, aspartyl-transfer RNA synthetase 2, arginyl-transfer RNA synthetase 2) also show recognizable phenotypes (Table 1). We suggest that a more thorough clinical characterization of patients may provide diagnostic clues, which may guide the otherwise inefficient candidate gene approach in combined respiratory chain deficiency. It is highly likely that numerous unknown disease genes are responsible for combined respiratory chain deficiencies. Recently, a novel method that uses a combination of bioinformatics, phylogenetic studies and homozygosity mapping (Pagliarini *et al.*, 2008) was proven to identify novel genes in complex 1 deficiency. This highly promising approach may be used to dissect the complex molecular mechanisms behind combined respiratory chain deficiencies.

In summary, thorough clinical characterization of patients with combined respiratory chain deficiency may help to identify homogeneous patient groups, implicating a defect in single common disease genes. This can be facilitated by linkage studies in large consanguineous families or in groups of similarly affected small families with similar clinical presentation. Functional cell culture investigations directed at different levels of the regulation of mitochondrial function (transcription, translation, ribosome function, protein stability, sub-complex formation) may provide a useful complementary tool in selecting novel candidates in combined respiratory chain deficiencies.

## Funding

Newcastle upon Tyne Hospitals NHS Charity (RES0211/7262 to R.H.); Academy of Medical Sciences, UK (BH090164 to R.H.); Wellcome Trust Senior Fellow in Clinical Science (to P.F.C.); Parkinson’s Disease Society (UK), the Medical Research Council Translational Muscle Centre and the UK NIHR Biomedical Research Centre in Aging and Age-related disease. Wellcome Trust and the MRC Centre for Translational Research in Neuromuscular Disease Mitochondrial Disease Patient Cohort (UK) (to R.W.T.); The Wellcome Trust (074454/Z/04/Z to R.N.L. and Z.M.A.C.L.), Biotechnology and Biological Sciences Research Council (BB/F011520/1 to R.N.L. and Z.M.A.C.L.) and the Medical Research Council (G0700718). The Muscle Tissue Culture Collection is part of the German network on muscular dystrophies (MD-NET, service structure S1, 01GM0601) funded by the German ministry of education and research (BMBF, Bonn, Germany). The Newcastle Biobank is part of the MRC Centre for Neuromuscular Diseases, UK. The Muscle Tissue Culture Collection is a partner of EuroBioBank (www.eurobiobank.org) and TREAT-NMD (EC, 6th FP, proposal # 036825). Mitochondrial diagnostic testing in Newcastle is funded by the UK National Commissioning Group to provide the ‘Rare Mitochondrial Disorders of Adults and Children’ service (http://www.mitochondrialncg.nhs.uk).

## Supplementary Material

Supplementary Data

## Abbreviations

COX: cytochrome *c* oxidase
SDH: succinate dehydrogenase
TRMU: 5-methylaminomethyl-2-thiouridylate methyltransferase
