# Supplementary material for: Nuclear factors involved in mitochondrial translation cause a subgroup of combined respiratory chain deficiency
Source: Brain. 2010 Dec 17;134(1):183–95. doi: 10.1093/brain/awq320 (PMC3707321; doi:10.1093/brain/awq320)
Supplement: Supplementary Data [file supp_134_1_183__index.html]

Nuclear factors involved in mitochondrial translation cause a subgroup of combined respiratory chain deficiency — Nuclear factors involved in mitochondrial translation cause a subgroup of combined respiratory chain deficiency — Supplementary Data 

# Nuclear factors involved in mitochondrial translation cause a subgroup of combined respiratory chain deficiency

## Supplementary Data

files

**Files in this Data Supplement:**

- Supplementary Data - docx file
