# Supplementary material for: Nuclear factors involved in mitochondrial translation cause a subgroup of combined respiratory chain deficiency
Source: Brain. 2010 Dec 17;134(1):183–95. doi: 10.1093/brain/awq320 (PMC3707321; doi:10.1093/brain/awq320)
Supplement: Supplementary Data [file supp_awq320_Supplementary_material_primers.docx]

**Intronic primers**

**MRPS16**

MRPS16-Exon1fw 5`-TGTAAAACGACGGCCAGTATTGTGGGAAGTATAGGGCG-3`

MRPS16-Exon1r 5`-CAGGAAACAGCTATGACCGTTCGGGTTGCCTCCATC-3`

MRPS16-Exon2fw 5`-TGTAAAACGACGGCCAGTGCCGTCTGTGTCATTTTGTC-3`

MRPS16-Exon2r 5`-CAGGAAACAGCTATGACCCCTACTGTCCTGGAGCTGAC-3`

MRPS16-Exon3fw 5`-TGTAAAACGACGGCCAGTGTTCCTAGGGAAGCATTGGC-3`

MRPS16-Exon3r 5`-CAGGAAACAGCTATGACCCCACACCCAGCTCTAAGTCAC-3`

| Exon 1 product size 328 bp |
| --- |
| Exon 2 product size 392 bp |
| Exon 3 product size 391 bp |

**TRMU**

TRMU-Exon1fw 5`-GCTACGGAGAGTCCGCC-3`

TRMU-Exon1r 5`- CTCCGAACCAAAGTCAGAGG-3`

TRMU-Exon2fw` 5`- CGTGTGGGGAACTTCTCAG-3`

TRMU-Exon2r 5`- ACCGTGGAGGGAGAGCG-3`

TRMU-Exon3fw 5`- AACAAATGTTCGATGACTGACG-3`

TRMU-Exon3r 5`-CTCTTGAGCCCAGGAGGTC-3`

TRMU-Exon4fw 5`- CAGCCCCTCAGCCTAAGAC-3`

TRMU-Exon4r 5`- AGTGATCCTCAGGCACAACC-3`

TRMU-Exon5fw 5`- CCTCTGACAGGCTAGGGGTAG-3`

TRMU-Exon5r 5`- GAAGGGGAAGTGGTGCG-3`

TRMU-Exon6/7fw 5`- CCTACCCTGGAAGCAAAGTG-3`

TRMU-Exon6/7r 5`- TTCCACCCTAGGCCGTG-3`

TRMU-Exon8fw 5`- TGAGTTACACCATTGCTGGG-3`

TRMU-Exon8r 5`- CTGGAGGCCTTCTCAAGC-3`

TRMU-Exon9fw 5`- TGGTAGGACAGTTGTTCCCAG-3`

TRMU-Exon9r 5`- CTTTCCTACCCCAGGGTCTC-3`

TRMU-Exon10fw 5`- CTGCCCTTCCCCTCTAAGC-3`

TRMU-Exon10r 5`-CCTCAGCCCCTTCCTCTG-3`

TRMU-Exon11fw 5`- GCTCAGTGCCTGGTGCTC-3`

TRMU-Exon11r 5`- TGCGTCTGCAGTCCACC-3`

| Exon 1 product size 760 bp |
| --- |
| Exon 2 product size 376 bp |
| Exon 3 product size 386 bp  Exon 4 product size 330 bp  Exon 5 product size 381 bp Exon 6/7 product size 493 bp Exon 8 product size 310 bp Exon 9 product size 332 bp Exon 10 product size 293 bp Exon 11 product size 702 bp |
|  |
